# Predicting Exact Valence and Arousal Values from EEG

**DOI:** 10.3390/s21103414

**Published:** 2021-05-14

**Authors:** Filipe Galvão, Soraia M. Alarcão, Manuel J. Fonseca

**Affiliations:** LASIGE, Faculdade de Ciências, Universidade de Lisboa, 1749-016 Lisboa, Portugal; fgalvao@lasige.di.fc.ul.pt (F.G.); smalarcao@ciencias.ulisboa.pt (S.M.A.)

**Keywords:** arousal and valence prediction, EEG, emotion recognition, comparative study

## Abstract

Recognition of emotions from physiological signals, and in particular from electroencephalography (EEG), is a field within affective computing gaining increasing relevance. Although researchers have used these signals to recognize emotions, most of them only identify a limited set of emotional states (e.g., happiness, sadness, anger, etc.) and have not attempted to predict exact values for valence and arousal, which would provide a wider range of emotional states. This paper describes our proposed model for predicting the exact values of valence and arousal in a subject-independent scenario. To create it, we studied the best features, brain waves, and machine learning models that are currently in use for emotion classification. This systematic analysis revealed that the best prediction model uses a KNN regressor (K = 1) with Manhattan distance, features from the alpha, beta and gamma bands, and the differential asymmetry from the alpha band. Results, using the DEAP, AMIGOS and DREAMER datasets, show that our model can predict valence and arousal values with a low error (MAE < 0.06, RMSE < 0.16) and a strong correlation between predicted and expected values (PCC > 0.80), and can identify four emotional classes with an accuracy of 84.4%. The findings of this work show that the features, brain waves and machine learning models, typically used in emotion classification tasks, can be used in more challenging situations, such as the prediction of exact values for valence and arousal.

## 1. Introduction

Emotions play an undeniably important role in human lives. They are involved in a plethora of cognitive processes such as decision-making, perception, social interactions and intelligence [1]. Thus, the identification of a person’s emotional state has become a need. Let us consider a scenario where we want to identify the emotional state of subjects from their EEG signals. However, we do not just want to identify whether a person is feeling positive or negative, or whether they are feeling a certain discrete emotion (e.g., happiness or disgust). We want more than that, we want to know the exact valence and arousal values that the person is feeling. This offers a wider range of emotional states and has the advantage that it can later be converted into discrete emotions if we wish.

**Figure 1:**
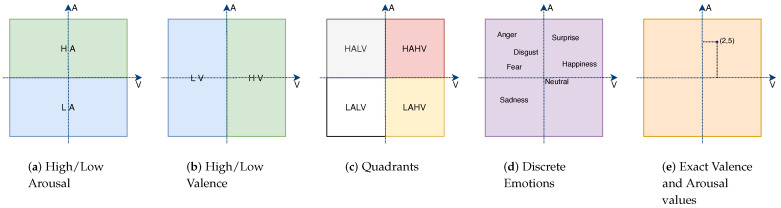
The typical set of emotions recognized in the literature (**a**–**d**) and what we want to achieve with our work (**e**). A: arousal; V: valence. (best seen in color).

There are several works that identify the emotional state of a person from EEG, as we discuss in Section 2, but the vast majority identify a small number of states, such as high/low valence and high/low arousal, or one of the quadrants of the circumplex model of affect (HAHV, HALV, LALV and LAHV, where H, L, A and V stand for high, low, arousal and valence, respectively). Thus, while several approaches for identifying discrete emotions have been proposed in the recent years, little attention has been paid to the prediction of exact values for valence and arousal (see Figure 1).

With this in mind, in this paper, we seek to answer to the following research questions: RQ1) Can EEG be used to predict the exact values of valence and arousal? RQ2) Are the typical features, brain waves and machine learning models used for classification of emotions suitable for the prediction of exact valence and arousal values? RQ3) Are the predicted valence and arousal values suitable for classification tasks with good accuracy?

To that end, we analyzed features from different domains (time, frequency and wavelet) extracted from the EEG signal, brain waves and machine learning methods for regression. For this purpose, we used three datasets (DEAP [2], AMIGOS [3] and DREAMER [4]) containing EEG signals collected during emotion elicitation experiments, together with the self assessment of the valence and arousal performed by the participants. We extracted time, frequency and wavelet features from EEG, considering alpha, beta and gamma bands, namely the three Hjorth parameters (activity, mobility and complexity), Spectral Entropy, Wavelet Energy and Entropy and IMF energy and entropy, as we describe in Section 3.

Experimental results, using a subject-independent setup with 10-fold cross-validation technique, show that our proposed model can predict valence and arousal values with a low error and a strong correlation between predicted and expected values (Section 5.2). Furthermore, in two subject-independent classification tasks (two classes and four classes), our model surpasses the state-of-the-art (Section 5.3).

Our main contributions can be summarized as follows:

- A systematic study of the best features, brain waves and machine learning models for predicting exact valence and arousal values (Section 4);
- Identification of the two best machine learning regressors (KNN and RF), out of seven, for predicting values for valence and arousal (Section 4.3);
- Combination and study of features from the time, frequency and wavelet domain, complemented with asymmetry features, for valence and arousal prediction (Section 4.4 and Section 4.5);
- A model able to predict exact values for valence and arousal with a low error, which can also predict emotional classes with the highest accuracy among state-of-the-art methods (Section 5).

## 2. Background and Related Work

To properly understand emotion recognition systems from EEG, we need to know: (1) the set of emotions to be detected and how they are modeled; (2) how EEG signals are related to emotions; (3) which brain waves and features best describe emotional changes in people; (4) which machine learning methods are most appropriate for emotion recognition.

### 2.1. Emotions

Emotions are generated whenever a perception of an important change in the environment or in the physical body appear. There are two main scientific ways of explaining the nature of emotions. According to the cognitive appraisal theory, emotions are judgments about the extent to which the current situation meets our goals or favors our personal well-being [5]. Alternatively James and Lange [6,7] have argued that emotions are perceptions of changes in our body such as heart rate, breathing rate, perspiration and hormone levels. Either way, emotions are conscious experiences characterized by intense mental activity and a certain degree of pleasure or displeasure.

There are two perspectives to represent emotions: discrete and dimensional. In the discrete perspective, all humans are thought to have an innate set of basic emotions that are cross-culturally recognizable. A popular example is Ekman’s six basic emotions (anger, disgust, fear, happiness, sadness and surprise) [8]. In the dimensional perspective, emotions are represented by the valence, arousal and dominance dimensions [9]. Valence, as used in psychology, means the intrinsic attractiveness or aversion of an event, object or situation, varying from negative to positive. Arousal is the physiological and psychological state of being awake or having the sensory organs stimulated to a point of perception, ranging from sleepy to excited. Dominance corresponds to the strength of the emotion. Dimensional continuous models are more accurate in describing a broader range of spontaneous, everyday emotions when compared to categorical models of discrete emotions [10]. For example, while the latter can only describe happiness, the dimensional representation can discriminate between several emotions near happiness, such as aroused, astonished, excited, delighted, etc. (Figure 1).

### 2.2. Physiological Signals and EEG

The use of physiological responses to characterize people’s emotional state has gained increasing attention. There are several physiological signals that can be used for this purpose, namely the electrical activity of the heart (ECG), galvanic skin response (GSR), electromyography (EMG), respiration rate (RR), functional magnetic resonance imaging (fMRI) or electroencephalography (EEG).

The latter provides great time resolution and fast data acquisition while being non invasive and inexpensive, making it a good candidate to measure people’s emotional state. The frequency of EEG measurements ranges from 1 to 80 Hz, with amplitudes of 10 to 100 microvolts [11]. Brain waves are usually categorized into five different frequency bands: Delta (δ) 1–4 Hz; Theta (θ) 4–7 Hz; Alpha (α) 8–13 Hz; Beta (β) 13–30 Hz; and Gamma (γ) > 30 Hz), each one being more prominent in certain states of mind. Delta are the slowest waves, being most pronounced during non-rapid eye movement (NREM) sleep. Theta waves are associated with subconscious activities, such as dreaming, and are present in meditative states of mind. Alpha waves appear predominantly during wakeful relaxation mental states with the eyes closed, and are most visible over the parietal and occipital lobes [12]. Beta wave activity, on the other hand, is related to an active state of mind, more prominent in the frontal cortex during intense focused mental activity [12]. Lastly, Gamma rhythms are thought to be associated with intense brain activity for the purpose of running certain cognitive and motor functions. According to the literature, there is also a strong correlation between these waves and different affective states [1].

### 2.3. Brain Waves and Features

One early decision when working with EEG for emotion recognition is related to the number of electrodes to use. In the literature, this number varies from only 2 electrodes [13,14] to a maximum of 64 electrodes [15,16], with the most common value revolving around 32 [2,17,18,19,20]. Usually, the placement of the electrodes in the scalp is done according to the international 10–20 system.

Another decision is related to the use of monopoles or dipoles. The former record the potential difference compared to a neutral electrode connected to an ear lobe or mastoid, while the latter collects the potential difference between two paired electrodes, thus allowing for the extraction of asymmetry features [16,19]. The asymmetry concept has been used in many experiments, and states that the difference in activity between the hemispheres reflects emotional positivity (valence). A left upper activity is related to a positive emotion (high valence), while a right upper activity is related to a negative emotion (low valence). According to the literature, the electrodes positioned in the frontal and parietal lobes are the most used because they have produced the best results.

Regarding brain waves, most researchers use the set comprised of theta, alpha, beta and gamma. Some also use the delta [15,21] or a custom set of EEG frequencies [22,23], while Petrantonakis et al. [24,25] used only alpha and beta frequencies, as these had produced the best results in previous works. The same for Zhang et al. [14,26], who used only beta frequencies.

Concerning the features to be extracted from the EEG, there is a great variety in the literature, with several authors using more than one type of feature extraction algorithm. The most used methods have been the Fourier Transform such as the Short-time Fourier Transform (STFT) or Discrete Fourier Transform (DFT) [16,27], statistical (mean, standard deviation, kurtosis, skewness, Pearson correlation) [19,28], Hjorth parameters (HP) [19,29,30], Power Spectral Density (PSD) [2,15,18], Wavelet Transform (WT) [15,31], Empirical Mode Decomposition (EMD) or Hilbert–Huang Spectrum (HHS) [24,26,27], Entropy such as the Differential Entropy (DE) [16,32], Approximate Entropy (AE) [15], Sample Entropy (SampEn) [26], Wavelet Entropy (WE) [15,31], Higher Order Crossings (HOC) [25], Fractal Dimensions (FD) [15,29,33], Auto Regressive Models (AR) [14] and Hurst Exponent (HE) [15]. When dipoles are used, the extracted features are called asymmetry measures. These include Differential and Rational Power Spectral Asymmetry (DPSA and RPSA) [2,18,34] or Mutual Entropy (ME)/Mutual Information (MI) [19]. It is also common to analyze the similarities between time series using Phase Coherence [19].

### 2.4. Emotion Classification

According to our literature review, almost all authors have used machine learning classifiers to recognize emotions. The most used have been the Support Vector Machines (SVM) [15,25,34], followed by K-Nearest Neighbors (KNN) [16,30,35]. Other classifiers used have been the Naive Bayes (NB) [2], Multi-Layer Perceptron (MLP) [34,35], Logistic Regression (LR) [16,32] and Random Forest (RF) [23,27]. In addition to these works that used hand-crafted features, there are other solutions that use deep-learning approaches, such as Artificial Neural Networks (ANN) [30], Deep Learning Networks (DLN) [18], Deep Belief Networks (DBN) [16], Long Short-Term Memory (LSTM) networks [36,37] or a combination of the latter with Convolutional Neural Network (CNN) [38,39].

The emotion classification problem has been done in one of three ways: (i) identification of discrete emotions such as happiness, scared or disgust [24,27,34,40,41,42]; (ii) distinction between high/low arousal and high/low valence [2,3,4,19,29,31,43]; and (iii) finding the quadrant, in the valence/arousal space [13,14,19,21,44,45]. In the last two cases, researchers create two classifiers, one to discern between high/low valence and the other for high/low arousal. Although binary classification is the most common, there are works in which researchers have performed multi-class classification [23,32]. There are also some works that included all positive emotions in one class and all negative emotions in another, and sometimes with the addition of the neutral class [15,16].

Table 1 summarizes the main characteristics of a subset of the reviewed papers, which include the database used, brain waves utilized, features extracted, classifiers employed and the set of emotions recognized. These works were chosen according to their relevance and novelty.

## 3. Materials and Methods

For creating our model, we explored different brain waves and features, and trained multiple regressors, using annotated datasets. Here, we describe all of them, plus the metrics used to evaluate the quality of the prediction.

### 3.1. Datasets

For our study, we used the AMIGOS [3], DEAP [2] and DREAMER [4] datasets, whose main characteristics are shown in Table 2.

The data used from the AMIGOS dataset that corresponds to the scenario where the 40 participants were alone watching 16 short videos: four in each quadrant of the circumplex model of affect. The EEG signals were recorded using the Emotiv EPOC Neuroheadset, using 14 electrode channels. The DEAP dataset contains data collected using 40 music videos, 10 in each quadrant. The EEG signal was recorded using 32 active AgCl electrodes with the Biosemi ActiveTwo system. The DREAMER dataset contains EEG signals recorded using the Emotiv EPOC Neuroheadset. Signals were collected from 23 participants while they watched 18 film clips selected to elicit nine emotions (amusement, excitement, happiness, calmness, anger, disgust, fear, sadness and surprise).

In the three datasets, participants performed a self-assessment of their perceived arousal, valence and dominance values using the Self-Assessment Manikin (SAM) [46]. In the case of DEAP and AMIGOS, participants also selected the basic emotion (neutral, happiness, sadness, surprise, fear, anger and disgust) they were feeling at the beginning of the study (before receiving any stimulus), and then, after visualizing each video.

### 3.2. Brain Waves

As we presented in the related work section, there is no consensus on which brain waves to use. However, considering the published results, we can see that the best accuracy is attained when using alpha, beta and/or gamma waves. Therefore, we studied only these three types of brain waves.

### 3.3. Features

The features analyzed in our work were selected based on their effectiveness, simplicity and computational speed, according to prior works, as described in Section 2. We studied the Hjorth parameters, Spectral Entropy, Wavelet Energy and Entropy and IMF energy and entropy.

#### 3.3.1. Hjorth Parameters

The Hjorth parameters [47] are obtained by applying signal processing techniques in the time domain, giving an insight into the statistical properties of the signal. The three Hjorth parameters are: activity, mobility and complexity (Equations (1)–(3)).

Activity gives a measure of the squared standard deviation of the amplitude of the signal x(t), indicating the surface of the power spectrum in the frequency domain. That is, the activity value is large if the higher frequency components are more common, and low otherwise. Activity corresponds to the variance of the signal.

(1) Activity=var(x(t))

Mobility represents the mean frequency or the proportion of standard deviation of the power spectrum. This is defined as the square root of the activity of the first derivative of the signal divided by the activity of the signal.

(2) $$\mathrm{Mobility}=\sqrt{\frac{\mathrm{Activity}(x^{\prime }\left(t\right))}{\mathrm{Activity}(x(t))}}$$

Complexity indicates how the shape of a signal is similar to a pure sine wave, and gives an estimation of the bandwidth of the signal. It is defined as the ratio between the mobility of the first derivative and the mobility of the signal.

(3) $$\mathrm{Complexity}=\frac{\mathrm{Mobility}(x^{\prime }\left(t\right))}{\mathrm{Mobility}(x(t))}$$

To summarize, the three parameters can be referred as the average power, the average power of the normalized derivative and the average power of the normalized second derivative of the signal, respectively.

#### 3.3.2. Spectral Entropy

Entropy is a concept related to uncertainty or disorder. The Spectral Entropy of a signal is based on Shannon’s entropy [48] from information theory, and it measures the irregularity or complexity of digital signals in the frequency domain. After performing a Fourier Transform, the signal is converted into a power spectrum, and the information entropy of the latter represents the Power Spectral Entropy of the signal [49]. Consider $x_{i}$ to be a random variable and $p(x_{i})$ its respective probability, then Shannon Entropy can be calculated as follows:

(4) $$H\left(x\right)=-\sum_{i=1}^{N}p\left(x_{i}\right)\;log_{2}\;p\left(x_{i}\right)$$

The Spectral Entropy treats the signal’s normalized power distribution in the frequency domain as a probability distribution, and calculates the Shannon Entropy of it. Therefore, the Shannon Entropy in this context is the Spectral Entropy of the signal if we consider $p(x_{i})$ to be the probability distribution of a power spectrum:

(5) $$p\left(x_{i}\right)=\frac{Psd(x_{i})}{\sum_{j}Psd\left(x_{j}\right))}$$

$Psd(x_{i}$ is the power spectral density, which is equal to the absolute value of the signal’s Discrete Fourier Transform.

#### 3.3.3. Wavelet Energy and Entropy

Wavelet transformation is a spectral analysis technique in which any function can be represented as an infinite series of wavelets. The main idea behind this analysis is to represent a signal as a linear combination of a particular set of functions. This set is obtained by shifting and dilating a single prototype wavelet ψ(t) called mother wavelet [50]. This is realized by considering all possible integer translations of ψ(t), and dilation is obtained by multiplying *t* by a scaling factor, which is usually a factor of two [51]. Equation (6) shows how wavelets are generated from the mother wavelet:

(6) $$\psi_{j,k}\left(t\right)=2^{j/2}\psi \left(2^{j}t-k\right)$$

where *j* indicates the magnitude and scale of the function (dilation) and *k* specifies the translation in time.

The Discrete Wavelet Transform (DWT) is derived from the continuous wavelet transform with a discrete input. It analyses the signal in several frequency bands, with different resolutions, decomposing the signal both in a rough approximation and detailed information. For this, it applies consecutive scaling and wavelet functions. Scaling functions are related to low-pass filters and the wavelet to high-pass filters [50].

The first application of the high-pass and low-pass filters produces the detailed coefficient D1 and the approximation coefficient A1, respectively. Then, the first approximation A1 is decomposed again (into A2 and D2) and the process is repeated, taking into consideration the frequency components of the signal we want to isolate [51]. Given that, in this work, we only consider alpha, beta and gamma frequencies, the number of decomposition levels used is three (D1–D3). Thus, D1 corresponds to gamma, D2 corresponds to beta and D3 to alpha. The mother wavelet chosen was db4, since it had already proven to generate good results in similar works.

Finally, after obtaining the detailed coefficients of the desired bands (decomposition levels) the Wavelet Energy can be computed by summing the square of the absolute value of these coefficients. The wavelet entropy can be calculated in a similar way to the Spectral Entropy.

#### 3.3.4. IMF Energy and Entropy

Empirical Mode Decomposition (EMD) is a data-driven method for processing non-stationary, nonlinear, stochastic signals, which makes it ideal for the analysis and processing of EEG signals [52]. The EMD algorithm decomposes a signal x(t) into a finite set of AM-FM oscillating components c(t) called Intrinsic Mode Functions (IMFs) with specific frequency bands. Each IMF satisfies two conditions: (i) the number of local extrema (maxima and minima) and the number of zero crossings differ by at most one; (ii) the mean value of the envelope defined by the local maxima and the envelope defined by the local minima is zero [52]. The general workflow of the EMD algorithm to decompose a signal is described in Algorithm 1.
**Algorithm 1** EMD decomposition steps.1:Identify all extrema (maxima and minima)2:Create the upper u(t) and lower l(t) envelopes by connecting the maxima and minima separately with a cubic spline curve3:Find the mean of the envelopes as $m\left(t\right)=\frac{u(t)+l(t)}{2}$4:Take the difference between the data and the mean: d(t)=x(t)−m(t)5:Decide whether d(t) is an IMF or not by checking the two basic conditions described above and the stoppage criterion6:If d(t) is not an IMF, repeat steps 1–5 on d(t) as many times as needed until it satisfies the conditions7:If d(t) is an IMF, assign it to an IMF component c(t)8:Repeat steps 1–7 on the residue, r(t)=x(t)−c(t), as input data9:The process stops when the residue contains no more than one extremum

After decomposition by EMD, the original signal x(t) is a linear combination of *N* IMF components $c_{i}\left(t\right)$ and a final residual part $r_{N}\left(t\right)$, as shown in Equation (7).

(7) $$x\left(t\right)=\sum_{i=1}^{N}c_{i}\left(t\right)+r_{N}\left(t\right)$$

EMD works as an adaptive high-pass filter, which isolates the fastest changing components first. Thus, the first IMFs contain information in the high frequency spectrum, while the last IMFs contain information within the lowest frequency spectrum. Since each component is band-limited, they reflect the characteristics of the instantaneous frequencies [53]. For this work, we focused on the first IMF, which roughly contains information within the gamma frequency range, the second IMF that contains the beta frequency spectrum, and the third IMF, which contains the alpha band [54]. To obtain the energy and entropy of the IMFs, we used the methods described in the previous sections.

### 3.4. Regression Methods

Since we intended to identify continuous valence and arousal values, the machine learning methods to be used should be regressors rather than classifiers. We studied seven methods: Linear Regression (LR), Additive Regression (AR), Decision Tree (DT), K-Nearest Neighbors (KNN), Random Forest (RF) and Support Vector Machines for Regression (SVR), with two kernels. These were chosen based on the analysis of the related work (see Section 2).

### 3.5. Metrics

To evaluate the regressors’ accuracy, we used three measures: the mean absolute error (MAE), Pearson correlation coefficient (PCC) and the root-mean-square error (RMSE) [55]. MAE measures the average magnitude of the errors in a set of predictions, without considering their direction. RMSE also measures the average magnitude of the error, but gives a relatively high weight to large errors. In our case, both metrics express the average model prediction error from 0 to 1. PCC measures the linear correlation between the ground-truth and predicted values. For MAE and RMSE the lower the value the better, while for PCC the closer to 1 the better. The formulas for each of the described measures are presented in Equations (8)–(10), where *y* represents a series of *N* ground-truth samples, and $\hat{y}$ a series of *N* predicted values.

(8) $$MAE=\frac{\sum_{i=1}^{N}\left|{\hat{y}}_{i}-y_{i}\right|}{N}$$

(9) $$RMSE=\sqrt{\frac{\sum_{i=1}^{N}{\left({\hat{y}}_{i}-y_{i}\right)}^{2}}{N}}$$

(10) $$PCC=\frac{N\sum_{i=1}^{N}\left({\hat{y}}_{i}y_{i}\right)-\sum_{i=1}^{N}{\hat{y}}_{i}\sum_{i=1}^{N}y_{i}}{\sqrt{N\sum_{i=1}^{N}{\hat{y}}_{i}^{2}-{\left(\sum_{i=1}^{N}{\hat{y}}_{i}\right)}^{2})}\sqrt{N\sum_{i=1}^{N}y_{i}^{2}-{\left(\sum_{i=1}^{N}y_{i}\right)}^{2})}}$$

## 4. Proposed Model

In this section, we describe both the process that led to the creation of the feature vectors, as well as the analysis performed to create our model.

### 4.1. Feature Vector

**Figure 2:**
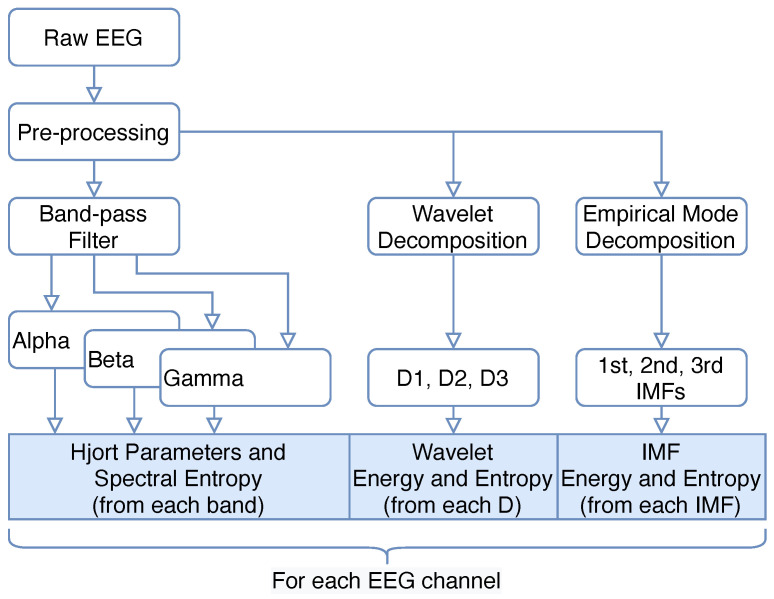
Steps to compute the feature vector from the raw EEG signal.

To compute the feature vector from the EEG signal, we started by performing a pre-processing step (Figure 2). Here, we first detrended the signal and eliminated the 50 Hz power line frequency by applying a notch filter. Then, to remove artifacts, we applied adaptive filtering techniques (for ECG artifacts) and wavelet thresholding (for EOG artifacts).

To extract the alpha, beta and gamma bands, we applied three FIR band pass filters to the EEG signal. Then, we computed the Hjorth parameters and Spectral Entropy for each of the bands. Wavelet and IMF-based features were calculated using the signal obtained after the pre-processing step, because these algorithms are band-limited.

We used a 4 s epoch, with 50% overlap, and computed the selected features for this window of the EEG. These values were selected based on the literature and after some preliminary tests.

### 4.2. Methodology

**Figure 3:**
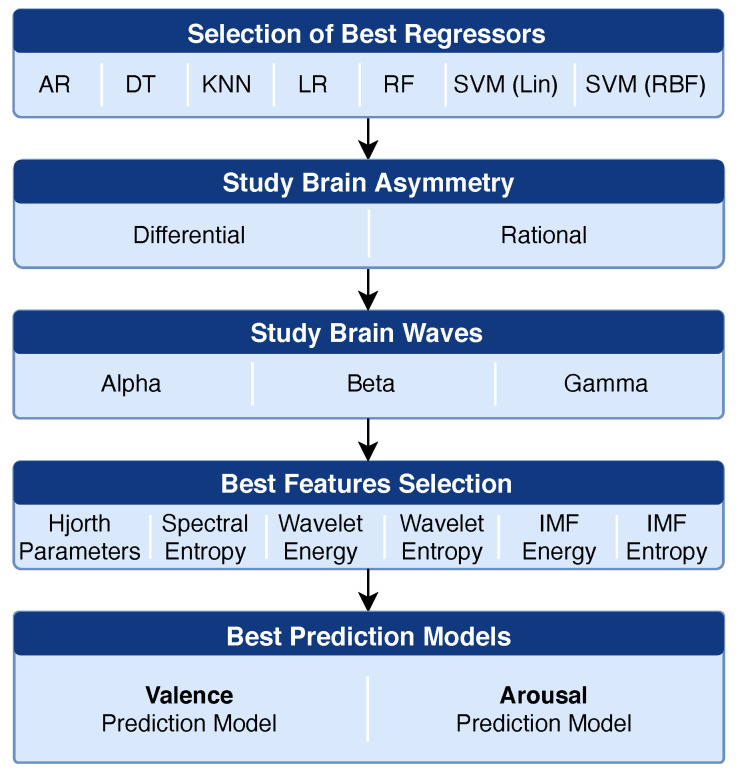
Steps of the analysis to identify the best configuration for our valence and arousal prediction models. (Lin: Linear; RBF: Radial Basis Function).

To create our model for valence and arousal prediction, we used the DEAP dataset as ground-truth, since it has been widely used and validated by the research community. To that end, we performed an analysis of multiple factors (regressors, brain asymmetry, waves and features) to identify the best configurations for our prediction models. The steps followed are represented in Figure 3 and described below:

1. Regressors Selection: In this step, we compared the accuracy of the seven regressors selected for our analysis. For that, we used a feature vector composed by all the features computed for all electrodes and all waves (but without the asymmetry features);
2. Brain Asymmetry: After identifying the best regressors, we checked if the use of brain asymmetry could improve the results, and if so, which type of asymmetry and waves produced the best results;
3. Features by Waves: We verified the accuracy achieved using each feature (for each brain wave) individually. To perform feature selection, we used a forward selection (wrapper method) [56], where we started by ranking the features based on their PCC values, and then added one by one to the model, until the results no longer improved;
4. Regressor Optimization: Finally, after identifying the best features, waves and regressors, we optimized the parameters of the selected regressors.

To train the several models on each step, we assigned to the feature vector extracted from each EEG epoch the self-reported values of valence and arousal present in the DEAP dataset. Since these values were reported for the overall video, all epochs from a video were annotated with the same valence and arousal values.

In all the experiments, we considered all the participants, and used a subject-independent setup through a 10-fold cross-validation. We randomly divided the dataset into 10 folds, then in turn, nine folds were used for training and the other one for testing. We report accuracy in terms of the three metrics (PCC, MAE, RMSE) as the average from the 10 iterations.

### 4.3. Regressors Selection

We started our analysis by identifying the best regression methods among the seven enumerated in Section 3.4: Additive Regression (AR), Decision Tree (DT), K-Nearest Neighbors (KNN), Linear Regression (LR), Random Forest (RF) and Support Vector Machine for Regression (SVR). The latter was tested with two different kernels, linear and Radial Basis Function (RBF)). We used the versions of these machine learning algorithms present in the Weka 3.8 software [57], with their default parameters.

We performed three tests for each regressor, one for each frequency band (alpha, beta and gamma). The feature vector used had a dimension of 256, composed by 8 features for each of the 32 channels: three Hjorth parameters (H1, H2, H3), Spectral Entropy (SE), Wavelet Energy (WP), Wavelet Entropy (WE), IMF energy (IMFP) and IMF entropy (IMFE).

As shown in Table 3, the RF and KNN regressors achieved the best results overall for the three bands. Although the DF presents better results than the KNN for the gamma band, it is worse for the other two bands. SVR, AR and LR present the worst results for all bands. As such, we selected RF and KNN (with K = 1) to be used in the remaining tests.

### 4.4. Asymmetry Features

The brain asymmetry concept has been widely used for emotion recognition, particularly for valence classification. Here, we tested two types of asymmetry: differential and rational. The former was calculated by considering the difference in feature values between homologous channels of opposing hemispheres (e.g., F3–F4, F7–F8, etc.). The rational asymmetry was calculated by dividing the feature values along the same homologous channels. The resulting feature vector, for both asymmetries, had a dimension of 112 (8 features for 14 asymmetry channels). We also combined the two feature vectors in a single one.

From Table 4, we can see that the differential asymmetry of the alpha waves produced the best results for the prediction of valence, using both regressors. The best results for arousal were achieved using a combination of both asymmetries of the gamma waves, using RF, and the rational asymmetry of the beta spectrum, using KNN. In both predictions (valence and arousal) the RF regressor achieved the best results.

### 4.5. Features by Waves

**Figure 4:**
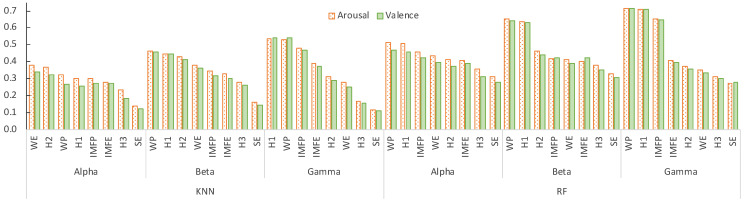
PCC values when each of the features per wave were used individually for predicting arousal and valence values. (best seen in color).

To identify the best features per wave (or the pairs wave-feature), we analyzed each pair individually, by training and testing a model for valence and another for arousal. Figure 4 shows the PCC values for the eight features per wave and for the two regressors. As we can see, the Wavelet Energy (WP) and the first Hjorth parameter (H1—Activity) produced the best results. On the other hand, the Spectral Entropy (SE) and the third Hjorth parameter (H3—Complexity) generated the worst results. Overall, beta and gamma features yield the best results for both regressors.

To identify the set of pairs wave-feature that produced the best values, we first ranked them by the PCC value, and then added one by one, starting from those with higher PCC values, and stopping when the inclusion of a new feature did not improve the results any further. We did this for each band separately and for band combinations, as well as using both regressors. The resulting set of waves and features for each combination is presented in Table 5.

Considering each wave alone, gamma and beta exhibit the best results in either regressor, for both valence and arousal. In general, KNN required more features than RF to achieve similar results, when considering single waves.

The combination of features from several waves improved the results, both for valence and arousal. For KNN, the combination of the three waves generated the best results, while for RF the addition of the alpha waves with other waves did not bring any improvement.

As we have seen in Table 4, the alpha differential asymmetry yielded the best results for valence prediction. Thus, we studied it to identify its best features. In Table 5, we can observe that the alpha differential asymmetry ($\alpha_{DA}$) generated much better results for valence than for arousal, as expected according to the literature.

Finally, we joined the alpha differential asymmetry with the best combination of waves. As we can see, we achieved the best results for arousal with this set of features. For valence, the values of PCC did not improve, but the MAE value for the KNN regressor was the smallest.

Before we selected the best features for each model, we performed a small test with the AMIGOS dataset. The test revealed that the combination of the alpha differential asymmetry with features from other waves yielded better results than using the asymmetry features only. Consequently, to attain a more generalized model, that is, one that would be accurate for all datasets with similar EEG characteristics and stimuli elicitation as the ones tested, we chose the following models for valence and arousal prediction:

- **KNN**: All features except the Spectral Entropy (SE) from the three bands, plus the alpha differential asymmetry, with all features except the third Hjorth parameter (H3). This yields a feature vector of dimension 770 for DEAP (7 features × 3 waves × 32 channels + 7 features from alpha waves × 14 asymmetry channels) and 343 for AMIGOS and DREAMER (7 features × 3 waves × 14 channels + 7 features from alpha waves × 7 asymmetry channels).
- **RF**: First Hjorth parameter (H1) and the Wavelet Energy (WP) from the beta and gamma waves, plus the alpha differential asymmetry from the first Hjorth parameter (H1), the Wavelet Energy (WP) and Wavelet Entropy (WE). The resulting feature vector has a dimension of 170 for DEAP (2 features × 2 waves × 32 channels + 3 features from alpha waves × 14 asymmetry channels) and 77 for AMIGOS and DREAMER (2 features × 2 waves × 14 channels + 3 features from alpha waves × 7 asymmetry channels).

### 4.6. Optimizing the Models

After identifying the best configuration for the four models, we performed a hyperparameter tuning to optimize the regressors. For KNN, we tested several K values (1, 3, 5, 7, 11 and 21), and used the Manhattan distance in the neighbor search instead of the euclidean distance. For RF, we tested 50, 500, 750 and 1000 trees (instead of the default 100).

From Table 6, we can see that KNN yielded the best results when K = 1. For RF, the results for 500, 750 and 1000 trees are equal for MAE and RMSE, and had a very small difference for PCC. Therefore, we opted for the 500 trees since it has a lower computational cost.

## 5. Experimental Evaluation

To assess the quality and generalization of the two models identified in the previous section, we conducted two experiments. One to evaluate the accuracy of the predicted values of valence and arousal, and another to assess the classification accuracy using the predicted values.

### 5.1. Setup

We conducted the evaluation using three datasets, DEAP, AMIGOS and DREAMER. For each dataset, we created the models using the settings identified in the previous section. In this way, we can understand how generalizable these settings are. We assessed the quality of the proposed models for two tasks: prediction and classification. In the former, we evaluated the models’ ability to predict the valence and arousal values, while in the latter, we measured the accuracy in identifying two classes (low/high arousal, low/high valence) and four classes (quadrants), using the estimated values to perform the classification tasks. In all the experiments, we used a subject-independent setup with a 10-fold cross-validation approach.

### 5.2. Prediction Results

As we can see in Table 7, both models (KNN and RF) achieved very good results for the three datasets, with PCC values greater than 0.755 and MAE values smaller than 0.158. In fact, and although the best models were found using the DEAP dataset, the quality of the prediction for the two unseen datasets (AMIGOS and DREAMER) is even better than for DEAP. This shows that the two identified models are generic enough to deal with new data.

Overall, results show that these models can predict valence and arousal with low error and a strong correlation with the expected values. The KNN presents the lowest errors (MAE) in all situations, while for PCC and RMSE both regressors present very similar values.

### 5.3. Classification Results

The final step of our evaluation consisted of evaluating the models in two subject-independent emotion classification tasks. One where we distinguish between low/high arousal and low/high valence (two classes), and another where we identify the quadrant in the valence/arousal space (four classes). To that end, we used the pair of predicted valence and arousal values.

#### 5.3.1. Arousal and Valence Binary Classification

In this classification task, we computed the accuracy rate for arousal and valence by averaging the classification rates for their low and high classes. We obtained these values for both regressors (KNN and RF) using the three datasets (see Table 8). As we can see, the KNN model achieved the best results for two datasets (DEAP and AMIGOS), while RF was slightly better than KNN from the DREAMER dataset. Thus, overall, we consider the KNN model to be the best one.

In Table 9, we compare the results achieved by the best identified model (KNN) with several recent works using the DEAP dataset. As we can see, our model achieved the highest classification rate, with values around 89.8% for both valence and arousal.

#### 5.3.2. Arousal and Valence Quadrants Classification

**Figure 5:**
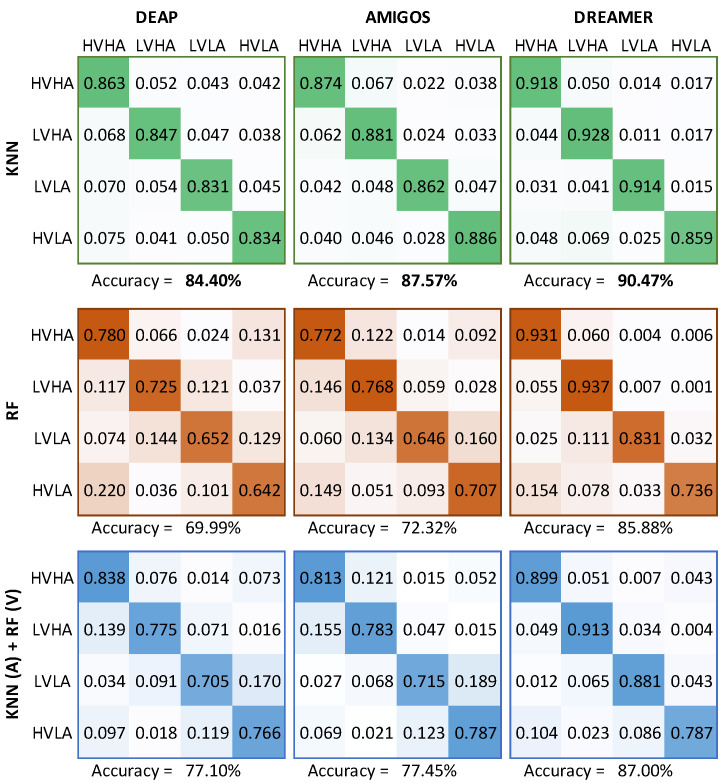
Confusion matrices of four quadrants classification for the KNN (**top**), RF (**middle**) and a combination of both regressors (**bottom**), using the datasets DEAP (**left**), AMIGOS (**center**) and DREAMER (**right**). (best seen in color).

In the second classification task, we identified the quadrant where the pair of predicted valence and arousal values was located. The classification results for KNN, RF and a mix of both (KNN for arousal and RF for valence due to the PCC values in Table 6) for the three datasets are shown in Figure 5. We present the results in the form of confusion matrices where rows represent the true quadrant and columns represent the predicted quadrant. It can be seen that the KNN-based models generated the best results for all datasets. This was foreseeable due to the small MAE values that these models displayed earlier (Table 6). We achieved better accuracy results for the two unseen datasets (AMIGOS and DREAMER), which shows that the features, brain waves and machine learning methods identified are generic enough to be used in unknown data.

Finally, we compared the classification results of the best identified model, with some recent approaches that perform a four class classification using the DEAP dataset. As we can see in Table 10, our best model (KNN) presents the best result, achieving an accuracy of 84.4%.

### 5.4. Discussion

The main goal of this work was to study the features, brain waves, and regressors that would ensure the creation of accurate and generalizable models for identifying emotional states from EEG, through the prediction of exact values for valence and arousal.

Our search for the best prediction models started with the comparison of several machine learning approaches, chosen based on their regular use and overall effectiveness and efficiency. RF and KNN achieved the highest PCC values and the lowest errors (MAE and RMSE), when compared to the remaining ones. Additionally, these regressors are relatively fast, making them good options for interactive applications where results should be produced in real-time.

The analysis of the features revealed that the first Hjorth parameter (H1), Wavelet Energy (WP) and IMF power (IMFP) generated the best accuracies on all frequency bands tested. These are features heavily correlated with power spectrum density. The other features, although not so relevant, also proved to be significant, as their inclusion in the KNN-based models improved the results. The only exception was the Spectral Entropy (SE), which whenever it was included deteriorated the results. The beta- and gamma-based features generated the best accuracies, which is consistent with the state-of-the-art.

The inclusion of the differential asymmetry of the alpha spectrum ($\alpha_{DA}$) improved considerably the valence prediction, as shown in Table 5. This corroborates the valence hypothesis, which states that the left hemisphere is dominant for processing positive emotions, while the right is dominant for negative ones [63].

After identifying the best features, we optimized the machine learning regressors by testing different values for their parameters (number of trees for RF, and K for KNN). For RF, we identified 500 trees, and for KNN, K = 1. We also changed the spatial metric of the KNN to the Manhattan distance, which improved the results.

To compare our results with previous approaches, we transformed the predicted valence and arousal values into high/low arousal and high/low valence (two classes) and the corresponding quadrant of the circumplex model of affect (four classes). In both classification scenarios, the identified KNN model achieved the highest accuracy, obtaining a value of 89.8% for two classes and 84.4% for four classes. These results are even more encouraging if we consider that they were obtained by predicting the arousal and valence values rather than directly from a classifier trained to identify classes (as the related work did). This means that we can accurately assess the emotional level of individuals by predicting arousal and valence values, and if necessary we can also identify discrete emotional classes.

From the achieved results, we can conclude that EEG can be used for predicting exact valence and arousal values (RQ1), and that the typical features, brain waves and machine learning models used for classification of emotions can be used for predicting exact valence and arousal values (RQ2). Finally, the two classification scenarios where we converted the predicted valence and arousal values into classes showed that our proposed model produces high quality results in classification tasks (RQ3).

## 6. Conclusions

In this work, we investigated the best combination of features, brain waves and regressors to build the best possible model to predict the exact valence and arousal values. We identified KNN and RF as the best machine learning methods for regression. In general, the features extracted within the beta and gamma frequencies were the most accurate, and the brain asymmetry concept of the alpha band proved to be useful for predicting valence. In the end, the KNN-based model, using all features except the Spectral Entropy, achieved the best accuracy for arousal and valence prediction, as well as for classification. A comparison with previous works, using the DEAP dataset, shows that the identified model presents the highest accuracies for two and four classes, achieving 89.8% and 84.4% respectively.

As future work, one can explore the use of these features and regressors in the analysis and classification of other physiological signals, since according to [64] entropy features in combination with RF showed good results for analyzing ECG signals.

## Figures and Tables

**Table 1:** A brief summary of the analyzed works and their main characteristics.

Work	Database	Features (Brain Waves)	Classifier	Emotions (#Classes)
[17]	MANHOB-HCI	PSD, DPSA (θ,α,β,γ)	SVM	arousal (3); valence (3)
[2]	DEAP	PSD, APSD (θ,α,β,γ)	NB	arousal (2); valence (2)
[41]	Video (Own)	SampEn, Spectral Centroid (α,β,γ)	KNN, PNN	disgust, happy, surprise, fear and neutral (5)
[18]	DEAP	PSD (θ,α,β,γ)	DLN, SVM, NB	arousal (3); valence (3)
[15]	Video (Own)	DPSA, WT, WE, AE, FD, HE (δ,θ,α,β,γ)	SVM	positive and negative (2)
[19]	DEAP	Pearson correlation, Phase coherence, MI (θ,α,β,γ)	SVM	arousal (2); valence (2)
[16]	SEED	PSD, DE, Differential/Rational asymmetry (δ,θ,α,β,γ)	KNN, LR, SVM, DBN	positive, negative and neutral (3)
[27]	DEAP	PSD, STFT, HHS, HOC (θ,α,β,γ)	RF, SVM	anger, surprise, other (3)
[29]	DEAP	Statistical, PSD, HP, FD (θ,α,β,γ)	SVM	arousal (2); valence (2)
[31]	DEAP	WP, WE (θ,α,β,γ)	SVM, KNN	arousal (2); valence (2)
[43]	DEAP, Music	PSD, FD, differential asymmetry (δ,θ,α,β,γ)	SVM, MLP, C4.5	arousal (2); valence (2)
[13]	MANHOB-HCI	EMD, SampEn (β)	SVM	high/low valence/arousal (4)
[14]	DEAP	EMD, AR (β)	SVM	high/low valence/arousal (4)
[4]	DREAMER	Logarithmic PSD (θ,α,β)	SVM	arousal (2); valence (2)
[3]	AMIGOS	Logarithmic PSD, APSD (θ,α,β,γ)	NB	arousal (2); valence (2)
[21]	DEAP	WP (δ,θ,α,β,γ)	ANN, SVM	high/low valence/arousal (4)
[44]	DEAP	Quadratic time-frequency distributions (Custom)	SVM	high/low valence/arousal (4)
[23]	DEAP, SEED	Flexible Analytic WT, Rényi’s Quadratic Entropy (Custom)	SVM, RF	high/low valence/arousal (4); positive, negative and neutral (3)
[42]	DEAP	PSD, APSD, Shannon Entropy, SE, ZCR, Statistical (θ,α,β,γ)	LSSVM (Least Square SVM)	joy, peace, anger and depression (4)
[45]	DEAP	WP, WE (θ,α,β,γ)	KELM	high/low valence/arousal (4)
[20]	DEAP, SEED	WT, High Order Statistics (δ,θ,α,β,γ)	DLN	high/low valence/arousal (4); positive, negative and neutral (3)
[22]	Video (Own)	LZC, WT, Cointegration Degree, EMD, AE (Custom)	SVM	arousal (2); valence (2)

**Feature Extraction:** AE—Approximate Entropy, APSD—Asymmetric Power Spectrum Density, AR—Auto Regressive models, DE— Differential Entropy, DPSA—Differential Power Spectral Asymmetry, EMD—Empirical Mode Decomposition, FD—Fractal Dimensions, HE—Hurst Exponent, HHS—Hilbert–Huang Spectrum, HOC—Higher Order Crossings, HP—Hjorth Parameters, LZC—Lempel-Ziv Complexity, MI—Mutual Information, PSD—Power Spectrum Density, SampEn—Sample Entropy, SE—Spectral Entropy, STFT—Short- Time Fourier Transform, WE—Wavelet Entropy, WP—Wavelet Energy, WT—Wavelet Transform and ZCR—Zero-Crossing Rate. Classifier: ANN—Artificial Neural Networks, DBN—Deep Belief Networks, DLN—Deep Learning Networks, KELM—Extreme Learning Machine with kernel, KNN—K-Nearest Neighbors, LR—Logistic Regression, MLP—Multi-Layer Perceptron, NB—Naive Bayes, RF—Random Forest and SVM—Support Vector Machines.

**Table 2:** Main characteristics of the AMIGOS, DEAP and DREAMER datasets.

	AMIGOS	DEAP	DREAMER
#Videos	16 (4 per quadrant)	40 (10 per quadrant)	18 (nine emotions twice)
Type	movie extracts	music videos	film clips
Duration	<250 s	60 s	65–393 s
Physiological Signals	EEG, GSR, ECG	EEG, GSR, BVP, RESP, SKT, EOG, EMG	EEG, ECG
Participants	40 (13 female)	32 (16 female)	23 (9 female)

**Physiological signals:** electroencephalography (EEG), galvanic skin response (GSR), electrocardiography (ECG), blood volume pulse (BVP), respiration (RESP), skin temperature (SKT), electrooculography (EOG) and electromyography (EMG).

**Table 3:** Results for each regressor per wave. Values in bold represent the two best results for each column in each wave. Grey rows represent the selected regressors.

		Arousal			Valence		
**Wave**	**Regressor**	**PCC**	**MAE**	**RMSE**	**PCC**	**MAE**	**RMSE**
Alpha	AR	0.248	0.205	0.245	0.172	0.224	0.264
	DT	0.339	0.194	0.241	0.263	0.216	0.261
	KNN (K = 1)	**0.459**	**0.158**	0.263	**0.417**	**0.178**	0.290
	LR	0.273	0.202	0.244	0.203	0.222	0.262
	RF	**0.517**	**0.177**	**0.219**	**0.480**	**0.197**	**0.238**
	SVR (linear)	0.251	0.200	0.248	0.232	0.237	0.242
	SVR (RBF)	0.244	0.212	**0.235**	0.214	0.211	**0.241**
Beta	AR	0.297	0.201	0.242	0.229	0.221	0.261
	DT	0.431	0.180	0.232	0.404	0.196	0.249
	KNN (K = 1)	**0.605**	**0.118**	**0.225**	**0.588**	**0.130**	0.244
	LR	0.301	0.200	0.242	0.280	0.216	0.257
	RF	**0.643**	**0.159**	**0.199**	**0.636**	**0.172**	**0.213**
	SVR (linear)	0.273	0.198	0.247	0.259	0.225	0.243
	SVR (RBF)	0.260	0.215	0.229	0.241	0.226	**0.239**
Gamma	AR	0.297	0.201	0.242	0.256	0.219	0.259
	DT	**0.472**	**0.173**	**0.227**	**0.486**	**0.183**	**0.237**
	KNN (K = 1)	0.409	0.174	0.275	0.387	0.191	0.297
	LR	0.247	0.203	0.246	0.255	0.218	0.259
	RF	**0.669**	**0.153**	**0.194**	**0.667**	**0.164**	**0.205**
	SVR (linear)	0.218	0.202	0.251	0.211	0.218	0.265
	SVR (RBF)	0.213	0.203	0.249	0.176	0.221	0.264

**Table 4:** Results for two types of asymmetry, and their combination, per wave. Bold values represent the best results for KNN and RF on each column.

			Arousal			Valence		
**Reg.**	**Asymmetry**	**Wave**	**PCC**	**MAE**	**RMSE**	**PCC**	**MAE**	**RMSE**
KNN	Rational	Alpha	0.410	0.175	0.275	0.366	0.196	0.302
		Beta	**0.558**	**0.132**	**0.238**	0.550	0.144	0.255
		Gamma	0.447	0.165	0.266	0.448	0.177	0.283
	Differential	Alpha	0.411	0.182	0.275	**0.739**	**0.125**	**0.191**
		Beta	0.338	0.195	0.290	0.337	0.209	0.308
		Gamma	0.271	0.212	0.305	0.279	0.227	0.322
	Both	Alpha	0.472	0.162	0.260	0.672	0.134	0.215
		Beta	0.530	0.141	0.245	0.525	0.152	0.262
		Gamma	0.422	0.172	0.271	0.423	0.185	0.289
RF	Rational	Alpha	0.516	0.176	0.218	0.481	0.196	0.237
		Beta	0.681	0.151	0.191	0.679	0.162	0.202
		Gamma	0.694	0.147	0.188	0.694	0.157	0.194
	Differential	Alpha	0.610	0.166	0.205	**0.862**	**0.120**	**0.153**
		Beta	0.674	0.151	0.192	0.672	0.162	0.203
		Gamma	0.689	0.148	0.189	0.685	0.158	0.200
	Both	Alpha	0.586	0.169	0.209	0.812	0.141	0.176
		Beta	0.680	0.150	0.191	0.682	0.161	0.202
		Gamma	**0.696**	**0.146**	**0.187**	0.694	0.156	0.198

**Table 5:** Results for the different features by waves, and their combinations. Values in bold represent the best results for KNN and RF, for valence and arousal. Grey rows represent the selected features.

		Arousal			Valence		
**Reg.**	**Waves & Features**	**PCC**	**MAE**	**RMSE**	**PCC**	**MAE**	**RMSE**
KNN	α(All−SE)	0.505	0.147	0.252	0.451	0.167	0.281
	β(All−SE)	0.621	0.114	0.221	0.597	0.126	0.241
	γ(WP+H1+IMFP)	0.535	0.139	0.244	0.528	0.149	0.260
	α,β(All−SE)	0.689	0.093	0.199	0.658	0.105	0.221
	α,γ(All−SE)	0.640	0.109	0.215	0.603	0.122	0.239
	β,γ(All−SE)	0.672	0.099	0.205	0.652	0.110	0.224
	α,β,γ(All−SE)	0.722	0.084	0.189	0.691	0.095	0.211
	$\alpha_{DA}\left(All\;-\;H3\right)$	0.430	0.178	0.270	**0.774**	0.115	**0.178**
	α,β,γ(All−SE)+ $\alpha_{DA}\left(All\;-\;H3\right)$	**0.743**	**0.078**	**0.182**	0.750	**0.082**	0.189
RF	α(All−SE−H3)	0.543	0.173	0.215	0.505	0.193	0.234
	β(WP+H1)	0.669	0.150	0.192	0.661	0.161	0.204
	γ(WP+H1+IMFP)	0.719	0.138	0.180	0.717	0.145	0.190
	β,γ(H1+WP)	0.722	0.138	0.180	0.719	0.147	0.190
	$\alpha_{DA}\left(H1\;+\;WP\;+\;WE\right)$	0.642	0.159	0.198	**0.901**	**0.097**	**0.126**
	β,γ(H1+WP)+ $\alpha_{DA}\left(H1\;+\;WP\;+\;WE\right)$	**0.748**	**0.136**	**0.175**	0.845	0.119	0.155

**Table 6:** Results for the optimization of the KNN and RF models, for different values of K, Manhattan distance and number of trees (T). Values in bold represent the best results for each model. Grey rows represent the selected parameters.

		Arousal			Valence		
**Model**	**Par.**	**PCC**	**MAE**	**RMSE**	**PCC**	**MAE**	**RMSE**
KNN α,β,γ(All−SE)+ $\alpha_{DA}\left(All\;-\;H3\right)$	K = 1	**0.794**	**0.062**	**0.163**	**0.795**	**0.066**	**0.172**
	K = 3	0.725	0.120	0.175	0.725	0.128	0.185
	K = 5	0.684	0.137	0.185	0.689	0.146	0.194
	K = 7	0.655	0.147	0.192	0.663	0.156	0.201
	K = 11	0.622	0.156	0.199	0.633	0.166	0.208
	K = 21	0.579	0.166	0.208	0.595	0.176	0.217
RF β,γ(H1+WP)+ $\alpha_{DA}\left(H1\;+\;WP\;+\;WE\right)$	T = 50	0.740	0.137	0.176	0.839	0.119	0.156
	T = 100	0.748	0.136	0.175	0.845	0.119	0.155
	T = 500	0.755	**0.135**	**0.174**	0.852	**0.118**	**0.153**
	T = 750	0.755	**0.135**	**0.174**	0.852	**0.118**	**0.153**
	T = 1000	**0.756**	**0.135**	**0.174**	**0.853**	**0.118**	**0.153**

**Table 7:** Prediction results using the datasets DEAP, AMIGOS and DREAMER.

		Arousal			Valence		
**Reg.**	**Dataset**	**PCC**	**MAE**	**RMSE**	**PCC**	**MAE**	**RMSE**
KNN	DEAP	0.794	0.062	0.163	0.795	0.066	0.172
	AMIGOS	0.830	0.045	0.129	0.808	0.063	0.175
	DREAMER	0.806	0.058	0.165	0.812	0.076	0.213
RF	DEAP	0.755	0.135	0.174	0.852	0.118	0.153
	AMIGOS	0.789	0.115	0.148	0.769	0.158	0.195
	DREAMER	0.864	0.099	0.142	0.870	0.128	0.181

**Table 8:** Accuracy values (%) for arousal and valence binary classification. Values in bold represent the best results for each column.

	DEAP		AMIGOS		DREAMER	
	Arousal	Valence	Arousal	Valence	Arousal	Valence
KNN	**89.84**	**89.83**	**92.46**	**90.69**	93.72	92.16
RF	80.62	85.91	85.98	83.00	**93.79**	**93.65**

**Table 9:** Comparison of the accuracy (%) of the proposed model with previous works, for arousal and valence binary classification (low/high arousal, low/high valence). Values are from the original papers and using the DEAP dataset.

Year	Method	Arousal	Valence
2020	Deep Physiological Affect Network (Convolutional LSTM with a temporal loss function) [36]	79.03	78.72
2020	Attention-based LSTM with Domain Discriminator [37]	72.97	69.06
2019	Spectrum centroid and Lempel–Ziv complexity from EMD; KNN [58]	86.46	84.90
2019	Ensemble of CNNs with LSTM model [39]	—–	84.92
2019	Phase-locking value-based graph CNN [59]	77.03	73.31
2018	Time, frequency and connectivity features combined with mRMR and PCA for features reduction; Random Forest [60]	74.30	77.20
2017	Transfer recursive feature elimination; least square SVM [61]	78.67	78.75
2012	EEG Power spectral features + Asymmetry, from four bands; naive Bayes classifier [2] (DEAP paper)	62.00	57.60
2021	Proposed model	89.84	89.83
	$\alpha ,\beta ,\gamma \left(All\;-\;SE\right)+\alpha_{DA}\left(All\;-\;H3\right)$; KNN, K = 1		

**Table 10:** Comparison of the accuracy (%) of the proposed model with previous works, for the four quadrants classification. Values are from the original papers and using the DEAP dataset.

Year	Method	Accuracy
2020	Nonlinear higher order statistics and deep learning algorithm [20]	82.01
2019	Wavelet energy and entropy; Extreme Learning Machine with kernel [45]	80.83
2019	Time-frequency analysis using multivariate synchrosqueezing transform; Gaussian SVM [62]	76.30
2018	Wavelet energy; SVM classifier [21]	81.97
2018	Flexible analytic wavelet transform + information potential to extract features; Random Forest [23]	71.43
2017	Hybrid deep learning neural network (CNN + LSTM) [38]	75.21
2016	Discriminative Graph regularized Extreme Learning Machine with differential entropy features [32]	69.67
2021	Proposed model	84.40
	$\alpha ,\beta ,\gamma \left(All\;-\;SE\right)+\alpha_{DA}\left(All\;-\;H3\right)$; KNN, K = 1	

## Short Biography of Authors

**Filipe Galvão** holds a master’s degree in Biomedical Engineering and Biophysics from the Faculty of Sciences, University of Lisbon (2020). His research interests include Emotion Recognition, Medical Signals Analysis, Signal Processing and Brain–Computer Interfaces.

**Soraia M. Alarcão** holds a master’s degree in Information Systems and Computer Engineering from IST/ULisbon (2014), and is currently a PhD student at the Informatics Department at Faculty of Sciences, University of Lisbon, Portugal. She is a researcher at LASIGE since 2014. Her research interests include Accessibility, Emotions Recognition, Human–Computer Interaction, Health Systems and Multimedia Information Retrieval.

**Manuel J. Fonseca** holds a PhD (2004) in Information Systems and Computer Engineering from IST/ULisbon, is an Associated Professor at Faculty of Sciences, University of Lisbon, and a senior researcher at LASIGE. His main research areas include Human–Computer Interaction, Emotions Recognition, Brain–Computer Interfaces, Multimedia Information Retrieval, Sketch Recognition and Health Systems. He is a senior member of IEEE and ACM, and a member of Eurographics.
